# Multifactorial hypophosphatemia: a diagnostic challenge involving denosumab, iron infusion, renal tubular acidosis, and gastrointestinal losses

**DOI:** 10.1093/jbmrpl/ziag121

**Published:** 2026-07-28

**Authors:** Liam Clifford, Tripti Joshi

**Affiliations:** Department of Endocrinology, Gosford Hospital, Gosford 2250, Australia; Department of Endocrinology, Gosford Hospital, Gosford 2250, Australia; University of Newcastle, Newcastle 2308, Australia

**Keywords:** hypophosphatemia, iron infusions, antiresorptive therapy, renal transplant, reduced bone mineral density

## Abstract

Hypophosphatemia is a complex electrolyte disturbance with diverse etiologies, including decreased intestinal absorption, increased renal loss, intracellular redistribution, and medication effects. We present a diagnostically challenging case of severe, persistent hypophosphatemia in a 39-yr-old female with a history of renal transplantation, chronic gastrointestinal disease, and osteoporosis. The patient presented with acute abdominal pain, nausea, and constipation and was found to have profound hypophosphatemia (<0.3 mmol/L), hypocalcemia, and hypokalemia. Her recent medical history included administration of i.v. ferric carboxymaltose and denosumab, both known to induce hypophosphatemia via FGF23-mediated renal phosphate wasting and secondary hyperparathyroidism. Additional contributing factors in her case included renal tubular acidosis, tertiary hyperparathyroidism, tacrolimus-induced tubular dysfunction, and significant gastrointestinal losses due to prior bowel resections and acute ileus. Despite aggressive i.v. and oral phosphate supplementation, hypophosphatemia persisted, necessitating prolonged therapy and multiple hospital admissions. Reduction of tacrolimus and correction of acidosis improved phosphate reabsorption, but recurrent episodes highlighted the multifactorial nature of her condition. This case underscores the importance of a systematic, multidisciplinary approach to diagnosing and managing hypophosphatemia, particularly in patients with overlapping risk factors and complex medical histories. Clinicians should maintain a high index of suspicion for delayed and persistent hypophosphatemia in at-risk populations especially following parenteral iron and antiresorptive therapies. Early recognition, regular monitoring, and individualized management are essential to prevent serious complications and improve patient outcomes.

## Introduction

Hypophosphatemia can result from decreased intestinal absorption, increased renal loss, phosphate shifts from the extracellular space to the intracellular space, or medication effects. Common causes include refeeding syndrome, hungry bone syndrome, antiresorptive therapy, osteomalacia, and Fanconi syndrome.^1^ Notably, certain i.v. iron formulations (iron polymaltose and ferric carboxymaltose [FCM]) can cause hypophosphatemia, typically developing 4-20 d after administration.^2–4^ Phosphate homeostasis is regulated by hormones such as PTH, FGF23, bone-derived factors, dietary and cellular factors, and 1,25(OH)2D3_._^5^ These influence intestinal absorption, renal reabsorption, bone resorption and mineralization, and cellular uptake and redistribution.^5^

Ferric carboxymaltose is the most common i.v. iron formulation associated with hypophosphatemia, occurring in >50% of recipients,^6^ and severe hypophosphatemia in 10%.^7^ Ferric derisomaltose is less commonly associated (5%).^6^ The hypophosphatemia from i.v. iron can persist for months.^8^ Symptoms range from mild (dizziness, weakness, and gastrointestinal disturbance)^6^ to severe (osteomalacia, seizures, cardiac and respiratory complications, and bone deformities).^2^^,^^9^^,^^10^ The mechanism involves FGF23-mediated phosphaturia and reduced vitamin D activity, leading to impaired phosphate absorption.^11^

Denosumab is an anti-RANKL Ab used in the treatment of osteoporosis. Side effects include hypocalcemia, localized inflammatory reactions, and rarely, rebound fractures of the spine.^12^ Uncommonly, hypophosphatemia has been reported following denosumab administration through secondary hyperparathyroidism and increased renal loss.^13^^,^^14^ Additional risk factors include poor dietary intake, gastrointestinal disturbance (chronic diarrhea, short bowel syndrome, and inflammatory bowel disease), medications (antacids, prednisolone, and phosphate binders), and misuse of alcohol.^15^

We report a case of severe hypophosphatemia following an i.v.-iron infusion, complicated by denosumab therapy for osteoporosis, prior renal transplantation, renal tubular acidosis (RTA), and gastrointestinal losses.

## Case report

A 39-yr-old female presented to the Emergency Department with a 2-d history of acute epigastric pain, nausea, and constipation. There was no weakness, myalgia, arthralgia, or paraesthesia. Her past medical history included meningococcal sepsis (complicated by cholecystectomy, splenectomy, finger amputations, and renal failure requiring a renal transplant), Clostridium infection with toxic megacolon requiring an ileostomy, multiple small bowel resections with adhesions and recurrent ileus, gastroparesis on home total parenteral nutrition (TPN), recurrent pancreatitis with type 3c diabetes mellitus, prior venous thromboembolism, tertiary hyperparathyroidism, osteoporosis, and superior vena cava stenosis. There was no history of fractures, alcohol use, or cigarette smoking. The first dose of denosumab 60 mg was administered 38 d, and i.v. FCM 500 mg 37 d, prior to this admission.

Regular medications included amitriptyline 10 mg daily, apixaban 5 mg twice daily (BD), cyclizine i.v. 50 mg ×5 times a day, Novomix 30/70 insulin 30 units with evening meal, mycophenolate 180 mg BD, olanzapine 5 mg daily, pantoprazole 40 mg daily, prednisolone 7.5 mg daily, rosuvastatin 20 mg daily, sulfamethoxazole-trimethoprim 400 mg-80 mg daily, and tacrolimus modified release 3.5 mg daily. The TPN formulation was Baxter Olimel N9E (PNS-3) which contained 30 mmol phosphate and was 1950 mL run over 15/24 h ×6 d a week.

**Figure 1:**
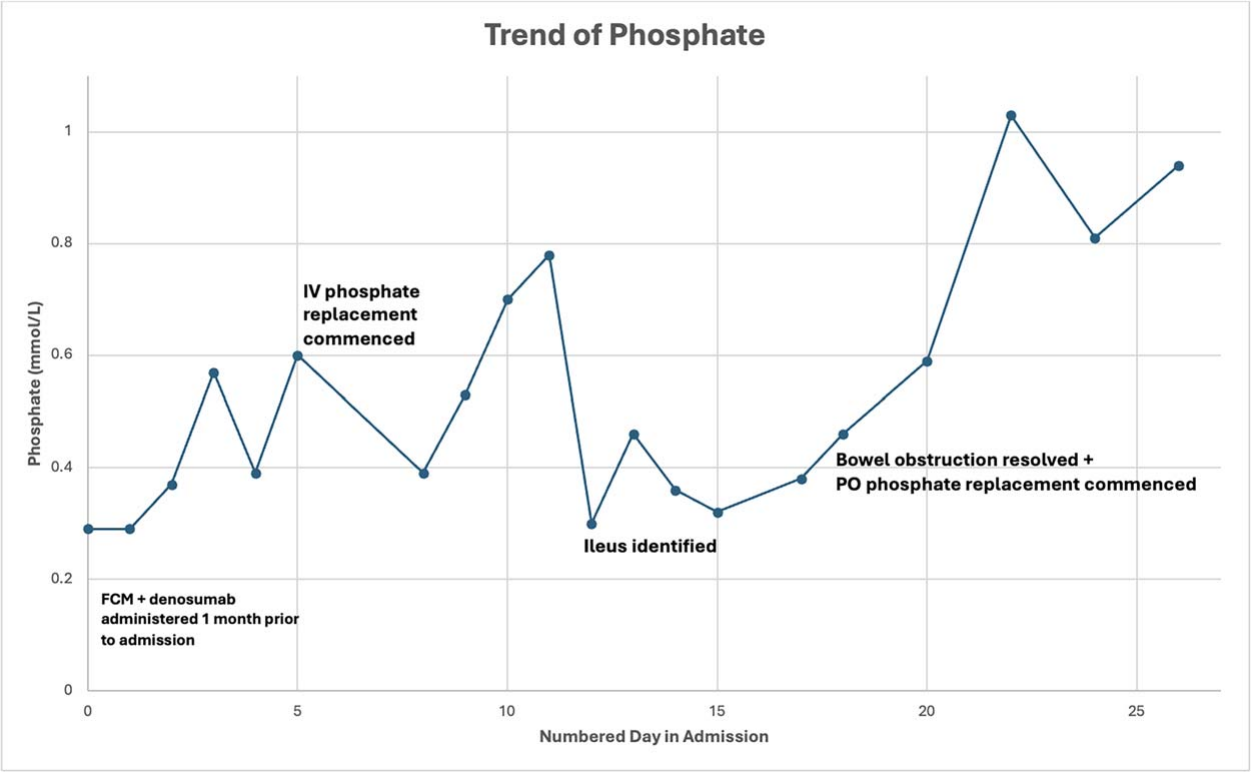
This is a graph demonstrating the trend of phosphate during admission.

Prior to admission the patient had iron studies performed which showed iron level 4 umol/L (reference range 5-30), transferrin 3.2 g/L (2.0-3.6), total iron-binding capacity 70umol/L (46-77), saturation 6% (10-45), and ferritin 16ug/L (15-200). She was administered FCM 500 mg 1 mo prior to admission. On admission, serum phosphate was <0.3 mmol/L (0.75-1.50), ionized calcium 0.95 mmol/L (1.15-1.30), corrected calcium 1.82 mmol/L (2.10-2.60), albumin 36 g/L (31-47), magnesium 0.65 mmol/L (0.70-1.10), potassium 2.9 mmol/L (3.20-5.30), blood urea nitrogen 5.6 mmol/L (3.0-7.0), creatinine 50 umol/L (45-90), and estimated glomerular filtration rate (eGFR) >90 mL/min/1.73m^2^ (>59). A “normal” FGF23 level of 30 ng/L (23.2-94.5) was identified. Lipase 15 units/L (10-60). PTH 130 pmol/L (1.6-7.2) with 25-hydroxyvitamin D 58 nmol/L (50-120). 24-h urine excretion of phosphate 132.9 mmol/L (12.9-33.0), calcium 13.1 mmol/L (1.50-7.50), and creatinine 6.6 mmol/L6.0-22.0) for a total urine collection of 4.7 L. Urinary magnesium was not measured. A diagnosis of mixed proximal and tubular dysfunction was made based on the following: Urine pH 6.5, serum pH 7.28 (7.35-7.45), serum bicarbonate 15 mmol/L (22-32), serum chloride 114 mmol/L (95-110), and an anion gap of 7 (4-12). Tacrolimus level was 3.2 ng/mL (5-15). Table 1 shows the trend of biochemistry. The trajectory of the phosphate is demonstrated in Figure 1. A CT scan of the abdomen was performed which demonstrated marked dilation of the small bowel loops which initally favored a diagnosis of ileus over mechanical obstruction, and the appearance of the pancreas was normal. The tubular maximum phosphate reabsorption per GFR (TmP/GFR) was 0.12 mmol/L (0.8-1.35). Venous blood gas results were consistent with a hyperchloremic normal anion gap metabolic acidosis. There was QT prolongation of 522 ms (<460) corrected for heart rate. No pathological signs were found on the chest X-ray.

**Table 1:** Trend in biochemistry.

Biochemistry	Reference range	Day 0 (Admission)	Day 3	Day 8 (ICU)	Day 12 (ICU)	Day 15 (ICU)	Day 26 (Discharge)
**Phosphate (mmol/L)**	0.75-1.5	<0.3	0.57	0.7	0.36	0.7	0.94
**Corrected calcium (mmol/L)**	2.1-2.6	1.82	1.99	1.71	1.95	2.21	2.18
**Magnesium (mmol/L)**	0.7-1.1	0.65	0.7	0.62	1.19	0.67	0.8
**Sodium (mmol/L)**	135-145	137	135	120	142	143	137
**Potassium (mmol/L)**	3.5-5.2	2.9	3.3	3.2	2.7	3.9	3.7
**Chloride (mmol/L)**	95-110	115	112	98	117	115	113
**Bicarbonate (mmol/L)**	22-32	11	17	14	16	16	14
**Serum pH**	7.35-7.45	7.28	7.29	7.30	7.34	7.34	7.33
**Urea (mmol/L)**	3-7	5.6	2.4	3.2	6.2	8.5	5.0
**Creatinine (umol/L)**	45-90	50	55	51	45	54	44
**eGFR (mL/min/1.73m** ^**2**^ **)**	>59	>90	>90	>90	>90	>90	>90
**25OHD (nmol/L)**	50-140	58	-	-	-	-	35
**Intact FGF23 (ng/L)**	23.2-95.4	-	-	-	-	30	-
**PTH (pmol/L)**	1.6-7.2	130	-	-	-	85	201

The patient received i.v. electrolyte replacement (including potassium dihydrogen phosphate 10 mmol and sodium dihydrogen phosphate 10 mmol). Due to ileus with reduced oral intake and ongoing gastrointestinal losses there was significant electrolyte disturbances which required regular replacement. The ileus was managed conservatively with a nasogastric tube and q4 hourly aspirates, but due to worsening gastrointestinal symptoms underwent a bowel resection for a volvulus at the level of previous enterocolic anastomosis, with resolution of symptoms subsequently achieved. The 25OHD level dropped to 35 nmol/L at time of discharge, likely reflecting a state of malnutrition with anorexia and chronic disease. After resolution, oral phosphate (sodium phosphate 1 g TDS) and high-dose vitamin D (50,000 units) were started. Despite treatment, hypophosphatemia persisted and relapsed, requiring prolonged supplementation (15 d of i.v. phosphate totaling 790 mmol and 3 d total of oral phosphate totaling 6 g). Reducing the tacrolimus dose improved renal biochemistry. Oral phosphate was discontinued at discharge, but recurrent hypophosphatemia has led to multiple subsequent hospital admissions despite the initial correction of phosphate in this index admission.

## Discussion

Severe hypophosphatemia is rare but can cause serious complications such as rhabdomyolysis, arrhythmias, seizures, and respiratory failure.^2^^,^^6^ In this case, persistent hypophosphatemia resulted from a combination of gastrointestinal losses, renal phosphate wasting, parathyroid dysfunction, vitamin D disturbance, and recent exposure to FCM and denosumab. This highlights the importance of considering multiple, overlapping causes and the need for a systematic, multidisciplinary approach to diagnosis and management.

Acute ileus, together with multiple prior bowel resections and adhesions, significantly reduced dietary phosphate intake in this case. As intestinal phosphate absorption is mediated primarily via intracellular mechanisms, this pathway is not affected by ileus. Hypophosphatemia frequently occurs in patients with malabsorption, bowel rest, or during refeeding.^16^ The TPN can help address absorption deficits, and delaying oral phosphate replacement until resolution of ileus is generally effective.^15^^,^^16^

Multifactorial renal phosphate wasting was a major contributor in this case, as evidenced by a markedly low TmP/GFR, indicative of a high FGF23 condition, and a lack of consistent correction with phosphate replacement. This very low TmP/GFR level highlights that the hypophosphatemia is being largely driven by renal wasting, supported by the presence of mixed type 1 and type 2 RTA. The patient’s prior renal transplant likely impaired regulation of FGF23 and PTH and contributed to tubular dysfunction.^17^ Immunosuppressive therapy, particularly tacrolimus, further exacerbated renal losses by contributing to a mixed RTA picture and reducing phosphate reabsorption.^18^ While prednisolone contributes to bone loss, its effect on phosphate wasting is not as profound as tacrolimus. Notably, reducing the tacrolimus dose and correcting acidosis led to significant improvement in phosphate reabsorption. Burosumab was not pursued as a treatment option as the patient did not qualify for a subsidized prescription.

Tertiary hyperparathyroidism, characterized by excess PTH, increases renal phosphate excretion via downregulation of NaPi-IIa and NaPi-IIc transporters.^19^ After renal transplantation, this condition can persist for years, leading to ongoing phosphate loss and reduced reabsorption.^17–19^ Concurrent vitamin D deficiency or low calcitriol further impairs intestinal phosphate absorption.^17^ Cincalcet was not pursued as a treatment option because the patient had a history of intermittent hypocalcemia secondary to gastroparesis, malnutrition, and multiple bowel resections. This case highlights the complexity of managing hypophosphatemia when multiple chronic pathologies coexist.

Antiresorptive therapies such as denosumab inhibit osteoclastic bone resorption, leading to increased phosphate release from bone.^17^ This can precipitate or worsen hypophosphatemia, especially in patients with vitamin D deficiency.^13^ It is increasingly more recognized that denosumab can worsen hypophosphatemia following an iron infusion.^20^ Although our patient’s vitamin D level was within the “normal” range, to exclude an effect of vitamin D insufficiency this level would ideally be above 75 nmol/L given her history of osteoporosis. Secondary hyperparathyroidism, marked by transient rises in PTH following antiresorptive therapy, further increases renal phosphate wasting.^14–19^ FGF23, secreted by osteoblasts and osteocytes, lowers serum phosphate; its levels rise after denosumab and iron infusions, peaking at 1-2 wk before normalizing more rapidly than phosphate stores are replenished.^6^^,^^12^ Notably, tertiary hyperparathyroidism and renal transplantation typically suppress FGF23.^19^ In our patient, an inappropriately normal FGF23 level likely reflected the interplay of mechanisms both promoting and inhibiting its production.

It has been hypothesized previously that iron infusions induce FGF23-mediated renal phosphate wasting by inhibiting FGF23 cleavage.^6–11^ Prolonged elevation of FGF23 after i.v. iron leads to persistent renal phosphate loss, making i.v. phosphate replacement ineffective for most patients. Oral replacement is preferred in chronic management, but this can be challenging in the setting of malabsorption or gastrointestinal dysfunction. Elevated FGF23 also inhibits 1α-hydroxylation of 25OHD; however, data suggesting it has a significant effect on phosphate metabolism are scarce.^12^ When denosumab and i.v. iron are administered in close succession, the risk of severe hypophosphatemia is significantly increased within the first month.^7–19^ These patients require ongoing oral phosphate and vitamin D supplementation, as replenishment of stores is slow, as demonstrated by our patient’s recurrent admissions for persistent hypophosphatemia. Regular monitoring of phosphate, calcium, and vitamin D in these patients would help initiate timely management.

This case illustrates severe disruption of phosphate homeostasis at gastrointestinal, renal, and skeletal levels. The combination of FCM and denosumab, alongside tertiary hyperparathyroidism, tacrolimus therapy, intestinal malabsorption, mixed RTA type 1 and type 2, and prior renal transplant, created a “perfect storm” of phosphate depletion that was resistant to standard replacement strategies. Such complexity underscores the importance of a systematic, multidisciplinary approach to diagnosis and management in patients with persistent hypophosphatemia.

## Conclusion

Severe hypophosphatemia can result from a combination of medication effects (such as parenteral iron and antiresorptive therapies) and underlying risk factors including gastrointestinal losses, renal phosphate wasting, parathyroid dysfunction, and vitamin D deficiency. Clinicians should be vigilant for delayed and persistent hypophosphatemia in at-risk patients, as it can lead to significant mortality. Early recognition, regular monitoring, and a multidisciplinary approach are essential to prevent and manage these complex electrolyte disturbances.

## Data Availability

Not applicable.
